# Producing knowledge together: a participatory approach to synthesising research across a large-scale collaboration in Aboriginal and Torres Strait Islander health

**DOI:** 10.1186/s12961-023-01087-2

**Published:** 2024-01-03

**Authors:** Kathleen P. Conte, Alison Laycock, Jodie Bailie, Emma Walke, Leigh-ann Onnis, Lynette Feeney, Erika Langham, Frances Cunningham, Veronica Matthews, Ross Bailie

**Affiliations:** 1 University Centre for Rural Health, The University of Sydney, Camperdown, Australia; 2 Oregon Health Sciences University-Portland State University School of Public Health, Portland State University, Portland, USA; 3 School of Public Health, The University of Sydney, Camperdown, Australia; 4 College of Business, Law and Governance, James Cook University, Cairns, Australia; 5 Poche Centre for Indigenous Health, University of Queensland, Brisbane, Australia; 6 Wellbeing and Preventable Chronic Diseases Division, Menzies School of Health Research, Charles Darwin University, Casuarina, Australia; 7 Sydney Medical School, The University of Sydney, Camperdown, Australia

**Keywords:** Knowledge translation, Indigenous health, Co-production, Collaboration, Systems thinking, Continuous quality improvement, Synthesis, Participatory research, Evidence review, Rapid synthesis

## Abstract

**Background:**

Despite that stakeholder participation in evidence synthesis could result in more useful outcomes, there are few examples of processes that actively involve them in synthesis work. Techniques are needed that engage diverse stakeholders as equal partners in knowledge co-production. The aims of this paper are to describe an innovative participatory process of synthesising a large body of academic research products and compare the findings of the participatory process against two traditional approaches to synthesis: a rapid review and a structured review.

**Methods:**

First, a rapid synthesis of all research outputs (*n* = 86) was conducted by researchers with in-depth knowledge of the collaboration’s research. Second, a team of researchers and service providers conducted a structured synthesis of seventy-eight peer-reviewed articles and reports generated by the collaboration. Fifty-five publications were brought forward for further synthesis in part three, a facilitated participatory synthesis. Finally, we explored the value added by the participatory method by comparing findings generated across the three synthesis approaches.

**Results:**

Twelve researchers and 11 service providers/policy partners—8 self-identified as Aboriginal and/or Torres Strait Islander—participated in two facilitated workshops (totalling 4 h). Workshop activities engaged participants in reviewing publication summaries, identifying key findings, and evoked review, discussion and refinement. The process explicitly linked experiential knowledge to citations of academic research, clearly connecting the two knowledge types. In comparing the findings generated across all three methods we found mostly consistencies; the few discrepancies did not contradict but gave deeper insights into statements created by the other methods. The participatory synthesis generated the most, detailed, and unique findings, and contextual insights about the relevance of the key messages for practice.

**Conclusion:**

The participatory synthesis engaged stakeholders with diverse backgrounds and skillsets in synthesising a large body of evidence in a relatively short time. The participatory approach produced findings comparable to traditional synthesis methods while extending knowledge and identifying lessons most relevant for the participants who, ultimately, are the end users of the research. This process will interest other large-scale research collaborations seeking to engage stakeholders in evidence synthesis.

**Supplementary Information:**

The online version contains supplementary material available at 10.1186/s12961-023-01087-2.

## Introduction

Participatory research aims to improve the quality and acceptability of research by including the intended beneficiaries in its creation and implementation [1–5]. “Who” participates, therefore, depends on the kind of research being produced and may include community members, policy makers, and people with lived experiences of the research topic [6]. Involving diverse perspectives as part of a research team provides a mechanism by which to incorporate multiple domains of scientific knowledge—experimental, observational, contextual, expert, and experiential [7]—into a research project; and therefore, improve the quality, relevance, uptake and impact of findings [1, 8]. Yet the process of engaging diverse perspectives can be challenging when participants’ worldviews, experiences, and research skills do not align [9]. While there are many examples of participatory research endeavours, there has been insufficient scholarship on how to best harness these diverse knowledges in the research production process [10]. Each collaboration is unique and contextual, and more examples of innovation around what “participation” could look like and how best to facilitate it, would be useful.

The primary aim of this paper is to describe a process of participatory knowledge translation and synthesis in the context of a large-scale research collaboration aimed at strengthening primary health care for Aboriginal and Torres Strait Islander peoples. Secondly, we compare the findings generated by the participatory process against findings generated via a more traditional, structured approach to synthesis and a rapid evidence synthesis of the same research to further explore the value of the participatory method.

### Participatory evidence synthesis

Engaging stakeholders in evidence synthesis is considered a best practice by Cochrane, the Campbell Collaboration, and others [11–14]. Yet there are few reviews that report using participatory approaches in evidence synthesis, and of those published still fewer describe how exactly stakeholders contributed [11, 15, 16]. In general, participation in research is limited to specific research stages—for example, in initial consultations, data collection and dissemination [14, 16–18]. There are relatively few examples that incorporate participation in evidence synthesis in spite of participants’ apparent interest being involved in analysis processes [19]. Barriers to involvement include concerns that specialized skills and training are required [19, 20]. We contend that participants’ existing skills and knowledge are considerations that should inform the design of participatory methods and not a limitation to surmount for participatory involvement.

Examples of projects that use participatory methods to help guide synthesis include participant engagement generally via workshops, and/or interviews occurring in parallel to the synthesis that then inform conceptual models [21, 22]; or, participant perspectives gathered after or iteratively with the synthesis to sense-check results and ensure the analysis is on track [23–25]. Researchers report pivotal insights provided by participation, including new conceptual ideas and explanations for program failures and successes [22–25]. In these examples, participant involvement operates alongside the analysis as a lens through which to view findings or as a guide to researchers who are creating findings as opposed to a mechanism by which to engage participants in developing findings themselves. But the synthesis process is arguably the most critical stage of the research process. It is the stage at which the new knowledge we set about to make is created. As the stage at which diverse perspectives can have the greatest impact in shaping knowledge, it is the most important stage to be participatory. Therefore, the onus is on researchers to make space and create opportunities where participants can contribute to analysis processes.

### Research context

This research took place within a long-standing Aboriginal and Torres Strait Islander health research collaboration. The Centre for Research Excellence in Integrated Quality Improvement (CRE-IQI) aimed to strengthen Aboriginal and Torres Strait Islander primary healthcare using continuous quality improvement (CQI) initiatives. CRE-IQI operated as an “innovation platform” by purposefully bringing together members representing multiple levels of the health system including researchers, policy officers, health service providers and practitioners and community members [26, 27]. Participation was fluid, with members engaging and participating in various ways at various times. CRE-IQI comprises Aboriginal and Torres Strait Islander people and non-Indigenous allies.

Throughout its operation, the collaboration maintained a strong program of integrated knowledge translation to ensure timely, culturally appropriate outputs for each project (see https://ucrh.edu.au/cre-iqi/, for a complete list of projects and resources and [28]). Towards the end of the CRE-IQI’s grant funding, the collaboration identified a need to synthesize the key findings of its research as a whole for dissemination, advocacy, and to inform future work. Between 2014 and 2019, the CRE-IQI produced numerous academic products including over 80 peer-reviewed articles, reports, and policy briefs and parliamentary submissions. We wanted to synthesise these products to produce a list of key messages for a final report [29] and series of policy briefs. Further, we recognized an opportunity to develop an innovative approach to synthesising research that would capture the experiential and contextual insights existing within the diverse collaboration. We were committed to developing a participatory process aligned with the CRE-IQI’s guiding motto: “All teach, all learn” [30] and the CRE-IQI’s guiding principles for practice. These principles included respecting the past and present experiences of Indigenous people, working in partnership, and establishing practices to support the application of evidence to improve Aboriginal and Torres Strait Islander primary healthcare and health outcomes [31].

### A systems-thinking orientation

We adopted a systems-thinking orientation to inform the participatory design described here. Systems thinking is a collection of theories, tools and practices that underlie a complexity informed approach to research and practice [21, 32–34]. It is a way of conceptualising social problems as adaptive, dynamic, interconnected, and intrinsically linked to human perception and experience [35]. Systems thinking emphasizes that no one person or perspective can fully understand a complex problem and so improvements come about through defining, sharing, and examining multiple perspectives and worldviews. In other words, collaboration and participatory processes are critical in knowledge generation.

Systems thinking is congruent with Aboriginal and Torres Strait Islander ways of knowing, being and doing. Many Indigenous knowledge systems are already centred on a holistic, systemic understanding of life, health, environment, and human connection [36]. Recently, Indigenous scholars have explicated linkages between Indigenous science and systems thinking as an emerging approach to complexity [37, 38], demonstrating the value of using systems thinking tools in this context.

We conceptualized the collaboration itself as a system whose purpose was to create new knowledge; a system in which each member, project, and activity provided a unique contribution to the process of knowledge production over time. The knowledge generated by the CRE-IQI was not solely contained in the written publications, but was created, embodied, and enacted among the members of the collaboration as they worked together to produce research. To use systems language, the knowledge generated by the CRE-IQI was “more than the sum of its parts.” This became our guiding ethos. We endeavoured to create a process that engaged members in the act of synthesis that drew both on the traditional academic research publications alongside members’ experiential knowledge. Our intention was to identify key messages from the research collaboration that embodied both.

## Methods

**Fig. 1:**
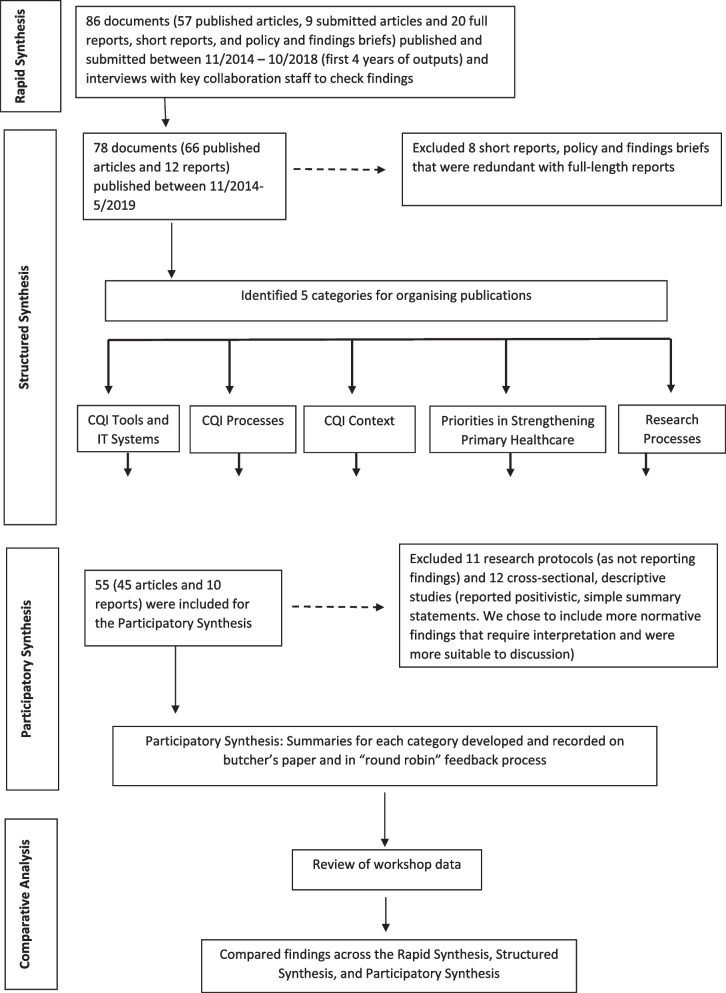
Flowchart depicting synthesis and analysis process

The research followed a multi-stage process, where the learnings and products from each stage were carried forward into subsequent stages (See Fig. 1 for an overview). First, a rapid review was conducted by CRE-IQI researchers who had extensive knowledge of the research outputs and findings. Then, we convened a diverse team of CRE-IQI members (hereafter, referred to as “Reviewers”) made up of Aboriginal and non-Indigenous colleagues, researchers, and service providers to collaboratively and systematically review the CRE-IQI literature, to prepare it for analysis in a participatory workshop with the broader CRE-IQI collaborative, and to facilitate that workshop. All the Reviewers are co-authors of this paper, and their details are provided in the authors’ note.

### Rapid Synthesis

In 2018, a Rapid Synthesis of CRE-IQI publications was undertaken as part of the CRE-IQI’s developmental evaluation. Three researchers (JB, AL, and RB) who had extensive knowledge of the CRE-IQI’s outputs and CQI literature compiled 57 published, peer reviewed articles, 9 submitted articles, and 20 full reports, short reports, and policy and findings briefs. They listed and organised the publications and identified key findings against the stated aims of the CRE-IQI for dissemination to the CRE-IQI network as part of an interim report on progress to date and to inform future directions. Subsequently, twenty-nine in-depth interviews with key stakeholders explored perspectives on the key findings from the rapid synthesis (more details about the evaluation and the interview findings are reported here [39, 40). The feedback provided a starting point and an initial organizing structure for the processes detailed below.

### Structured Synthesis

In March–May 2019, we conducted a structured review and synthesis of all published CRE-IQI publications and full-length reports. The inclusion criteria were: (1) published between November 2014 and May 2019; and (2) identified by the author(s) as CRE-IQI research. We excluded 8 short reports and policy and findings briefs identified in the Rapid Synthesis because they were redundant with full-length reports already in the sample. This resulted in 78 publications (66 peer-reviewed articles and 12 reports), all of which were included in the full-text review.

The review process was designed to systematically extract key characteristics of the publications while simultaneously familiarising Reviewers with the CRE-IQI literature so they could lead group discussions during the Participatory Synthesis (described below). To ensure consistency, we developed a template to guide data extraction into a spreadsheet. We extracted characteristics of the research reported—e.g. study design, topic area, measures used, etc.—as well as findings related to the five aims of the CRE-IQI as detailed in the original grant application.

Using the aim or purpose statement of each publication, NVIVO [41] (a qualitative data management tool) was used to help sort the publications into initial categories that aligned with aims of the CRE-IQI. Using this initial organizing scheme, reviewers extracted data into a spreadsheet from thematically similar publications. To provide consistency and a quality check, each publication was reviewed by at least two Reviewers and discrepancies were resolved by a third. Summarized publication characteristics, (i.e., by context, topic area, study design, alignment with CRE-IQI aims), were developed from the extracted material (a complete list of findings statements is available in Additional file 1). We developed brief descriptions of each publication for use in the Participatory Synthesis. We also revised the organizing categories (reported in Fig. 1) because we found that research foci had evolved over time and that organizing by CRE-IQI aim did not fully and evenly represent the content of the publications.

Of the 78 documents reviewed, 55 (45 articles and 10 reports) were brought forward to the Participatory Synthesis. Excluded publications included 11 research protocols that did not report research findings and a group of 12 cross-sectional, descriptive studies that reported on prevalence, rates of screening, follow-up care, and variations in practice for a range of health issues in Aboriginal and Torres Strait Islander primary health care (see Fig. 1). We excluded the latter publications because the findings were positivistic in nature—i.e., descriptive statements of statistical findings lending themselves to simple, declarative, summary statements (e.g., “The proportion of eligible patients with documented cardiovascular risk assessment was 33% (*n* = 574/1728)” [42]). We decided to focus the Participatory Synthesis on findings that were more normative in nature and would require a greater degree of interpretation on the part of the reader, and therefore, discussion on the part of synthesis participants.

### Participatory Synthesis

Interactive workshop sessions were hosted as part of a 2-day Biannual CRE-IQI meeting. The workshops aimed to identify the key learnings from CRE-IQI publications, with the Reviewers identified in the previous process acting as conversation “hosts” for discussion groups. A detailed activity guide for the participatory workshops is provided in Additional file 2.

Twenty-three workshop participants (12 = researchers; 11 = service providers/policy partners; 8 of whom identify as Aboriginal and/or Torres Strait Islander) were given the list of five revised organising categories and self-selected into five groups for a 2.5-h workshop. Each group took on one category of publication (e.g., ‘CQI processes’). We encouraged a mix of researchers and service providers in each group to ensure diverse perspectives.

Within each group, we asked researchers and service providers to work in pairs or threes to review the publication summaries, discuss, and select the main or most important finding(s) as related to their category. They wrote these findings on slips of paper, along with the number assigned to the publication summary so that each finding was explicitly linked to a publication. The Reviewers provided guidance to facilitate the activity and provide additional information about the publication contents if requested to aide interpretation.

**Fig. 2:**
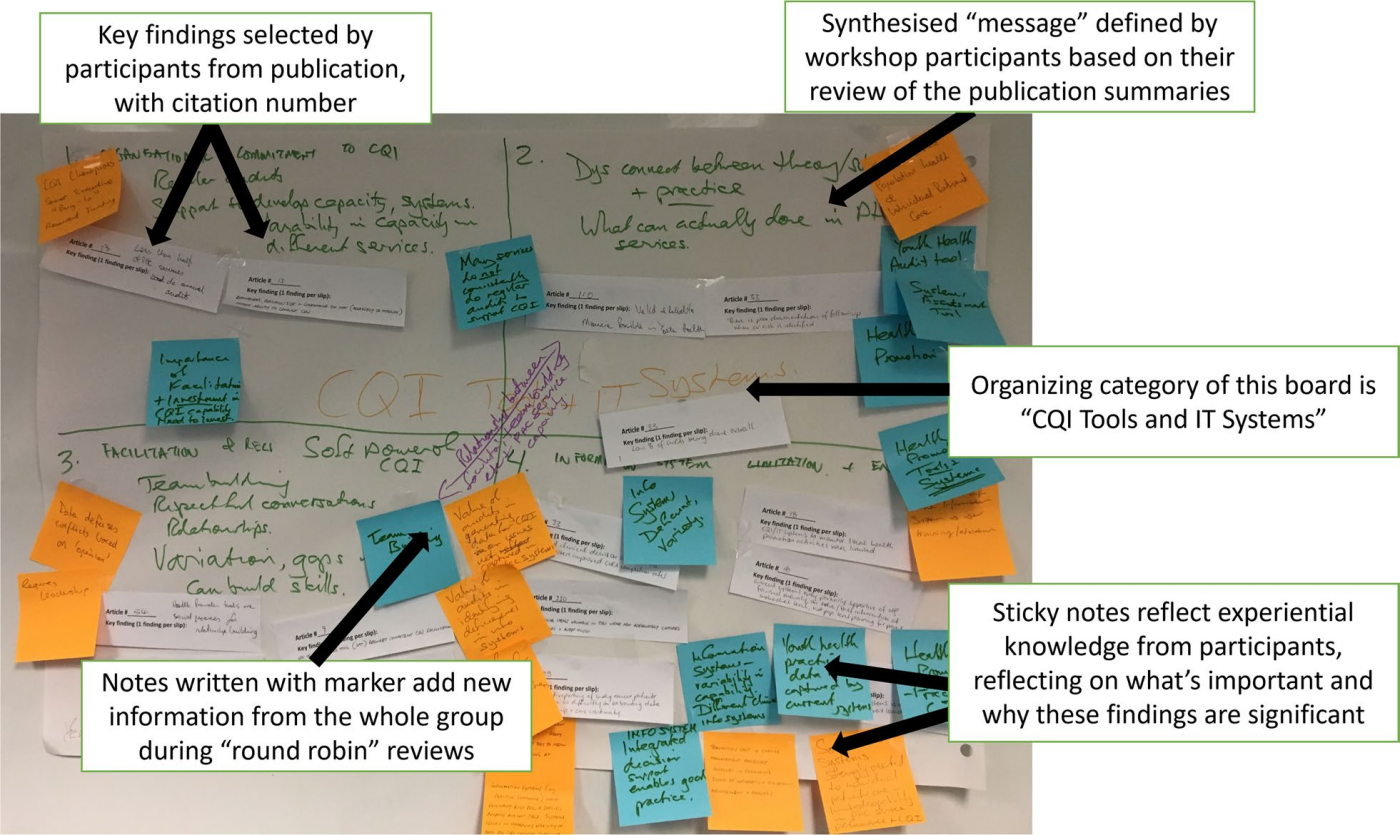
Example of data from Participatory Synthesis

Guided by the Reviewers, each group then sorted the findings and developed descriptive statements of the findings, or key messages. We asked participants to develop labels describing what each grouping of findings was about, and to constantly reflect on what the most important findings were and why. We asked them to think about their personal/professional experiences to help explain why findings were meaningful and to write these insights on post-it notes and add them to the growing documentation on butchers’ paper (see Fig. 2).

The next day, in a 1.5 h session and using a “round robin” methodology [43], the five groups reviewed each other’s work. Reviewers stayed with their group’s butchers’ paper poster and presented a summary of findings and insights to each visiting group. Then, participants took turns reacting to and adding new information using markers and sticky notes. They were prompted to add: (a) citations from the CRE-IQI publications that they reviewed that also aligned with the statements, and (b) insights from their own knowledge or experience that helped explain the importance or significance of the findings. After about 10 min, each group would move to the next category thus providing every participant an opportunity to review, add to, and provide clarifications to the findings and messages for each category of publications.

At the end of the process the data produced included: audio recording of each groups’ discussion on day 1; subcategories within each organizing category with specific findings and the publications linked to each point; and sticky notes that provided additional, experiential insights into the significance and relevance of each finding from days 1 and 2 (See Fig. 2).

Two authors (KC and AL) met and reviewed the data produced from the workshop. We reviewed the audio recordings and noted additional details from groups’ conversations that provided clarifications to their written findings or captured insights that were not recorded in writing. We referred frequently to the synthesis extractions and original publications to verify findings captured by participants and to identify any new content added by the participants during the synthesis process. We were able to ‘member-check’ the findings and messages at a subsequent biannual meeting of the CRE-IQI.

An evaluation of the entire biannual meeting was conducted; however, it was not specific to the participatory analysis activities. Therefore, after the meeting we emailed Reviewers and participants to request written feedback on the Participatory Synthesis process.

## Comparative analysis of three synthesis approaches

As we designed the collaborative and participatory processes, we realized our context offered an opportunity to compare findings statements about CRE-IQI research from three different approaches to evidence synthesis. As previously described, we used a rapid review to initially review and organize the literature, followed by a structured, systematic-type approach to extract key information and findings and summarize the literature against the five aims of the CRE-IQI; and a participatory approach to involve participants in identifying key findings from publications. As systematic and rapid reviews constitute the dominant review types in academic literature, a comparison of these traditional approaches with a participatory approach enables reflection on the additional value and/or weaknesses the participatory method adds.

We compared the findings statements generated by the three approaches described above using a process inspired by Betzner et al. [43]. Betzner and colleagues used three evaluation methods in a smoking cessation study, analysed each independently, and then combined the analyses to identify convergent, divergent, and unique findings. In our study, findings from each synthesis type were collated and given a unique number. In a table, findings were organized and matched to each other by subject (row), and by synthesis type (column). Where a statement contained more than one finding, the statements were broken down so that each finding could be analysed on its own. Matched findings were labelled “Strong agreement”, “Moderate agreement/disagreement”, “Disagreement” or “Unique”. For example, one statement in the Participatory Synthesis stated the importance of adapting continuous quality improvement (CQI) approaches to each unique context to ensure that CQI processes are responsive, contextually relevant, and participatory. While the particular statement reflected CQI theory and practice, these elements were not identified in the Rapid or Structured Synthesis and so considered “Unique”.

In total, there were four possible comparison outcomes across each synthesis type, between: (1) the Rapid Synthesis and Structured Synthesis, (2) the Structured Synthesis and Participatory Synthesis, (3) the Participatory sSynthesis and Rapid Synthesis, and (4) all three. A scoring system was developed to track the number and types of agreements for each finding so that agreements could be summed and compared across synthesis types and by content area (see Additional file 3 for a table reporting the scoring results). Where there was strong agreement, high-level statements were created that summarized key findings across the methods. Findings with moderate agreement were examined in more detail to develop statements that summarized the discrepancy between findings.

## Results

### Outcomes of the Participatory Synthesis

Participants of the Participatory Synthesis were highly engaged in conversation about the findings and about their own experiences. Participants new to the collaboration and those who were not embedded in the research settings seemed comfortable in engaging with the research findings and considering the relevance to their own practice. Four Reviewers and 5 participants responded to our request for written feedback via email after the workshop. Overall, the responses were positive and emphasised the value of engaging all members in the process (See Table 1 for feedback examples). Respondents offered insights into key features of the design that made the process work successful, for example, the “round robin” activity to review and reflect on the initial findings and the importance of the Reviewers' familiarity with the work. Some respondents reflected that some researcher participants struggled to see this experience as a scientifically valid or rigorous process.

**Table 1:** Selected feedback on the Participatory Synthesis process from reviewers/conversation hosts and participants

*Feedback from reviewers/conversation hosts*
• I really enjoyed this activity. I think that the fact that it took some effort to move groups on to the next poster suggests that others enjoyed it too. The discussion about the content of the poster and the sharing of information as we moved around the room was really interesting… Important for the facilitators to have read the articles and summaries—this helped to get the best from the pairs of researchers/service providers … [It was] remarkably difficult to get researchers to move on to higher level synthesis of info—they tend to want to review all the detail themselves, reluctant to trust and take others’ perceptions at face value, and to work with the synthesis of these perceptions rather than go back to the specifics of the papers. (Reviewer 1) • The [second workshop] helped people to process the info; it was useful for teams to come back to this after a break, and to hear others’ perceptions, questions. (Reviewer 2) • I think it worked well—I thought that the way it was rolled out was excellent—clear and precise instructions at each stage made the sessions run well. (Reviewer 3) • I think that it did help to have someone in the group who had read all the articles ahead of the session. It will be interesting to see how it all comes together…I think you did well with structuring the whole approach which has been very interactive and novel….I think that it was a useful process to have the wider review by all participants/groups of the workings from the first session. It was helpful for our group, because this process allowed for service provider representatives to review our work in the first session, and to add/supplement the initial work. (Reviewer 4)
*Feedback from participants*
• For me the highlight was the small group discussions. They provide opportunity to share ideas from both sides, particularly when the group consists of a mix of professionals with different areas of expertise. The session on analysing the published CRE literature was particularly beneficial for me, even though there were perceptions that it was a superficial experience. Once we extracted the key information and then sorted them it was exciting to see some common themes coming up. I felt very proud to be part of a team that has done such wonderful work. (Participant 1) • It was refreshing in the sense of being an active interactive process. It got people standing and moving rather than the usual sitting for a long time. [A benefit was] An overview of the scope and key elements of the CQI related research findings… I think the approach works and will be useful to apply in other contexts. (Participant 2) • As part of the [Developmental Evaluation] process it is important to try new things and this workshop approach is a brilliant idea—it’s a different way of getting more from research results and a way of getting a ‘global’ perspective, a helicopter view of the CRE research findings—I really liked the concept, a great idea. (Participant 3) • As an exercise for CRE, the workshop is not so much about having a scientifically rigorous research process that can be validated but as an internal process for giving voice to all members of the CRE. It is a perfectly good way to bring all voices together to sift out what should be the key messages. I think it is a really great way to give people ownership and to be able to participate in that process. It is an organisationally sound, useful, engaging, collaborative process for doing something that could otherwise be done by a select few. (Participant 4)

CQI - Continuous Quality Improvement; CRE - Centre for Research Excellence [in Integrated Quality Improvement]

A few researchers in the workshop voiced concerns about the possibility of the participatory process yielding “cherry picked” results. The ensuing discussion concluded that audiences will always seek results that provide insights to what they are interested in knowing or answering so that the bias present in the participatory process is no greater than that of usual consumption of research. There was also a strong expectation from stakeholders that each findings statement in the final report be accompanied by a citation despite them being synthesized from multiple forms of knowledges.

In response to these concerns and preferences, we re-reviewed the publications to establish whether the “new” findings generated by the Participatory Synthesis could be supported by CRE-IQI evidence and were able to validate that all statements generated from the Participatory Synthesis were supported by statements in the publications, even when the publications were not provided to the participants at the workshop.

After the participatory workshop, we produced overarching findings statements and a "Visual Bibliography". Using metaphor and visualisations alongside citations, the Visual Bibliography provides an artistic “map” of the CRE-IQI’s complex body of research. Research topics and key messages are artistically depicted alongside reference numbers that guide the viewer to the publication either by a direct web-link, or the attached reference list. The development, purpose and design of the Visual Bibliography is described in detail elsewhere (paper currently under review [28]). Both products were presented in draft form to participants at a follow-up workshop several months later which enabled refinement. The final statements and map were presented in the final report of the CRE-IQI [29]. In addition to producing an output for future dissemination, the process itself was a form of knowledge translation where participants learned more about the research findings while completing the workshop.

## Outcomes of the comparative analysis

Seventy-eight findings statements were developed from the three synthesis methods: 26 from the Structured Synthesis, 35 from the Participatory Synthesis, and 17 from the Rapid Synthesis (Table 2 list of statements by synthesis type). Most of the statements were either in agreement across two or more of the synthesis methods (*n* = 15), or unique to one method (*n* = 21). There were no instances of disagreement (see Additional file 3 for details). Most agreement was observed among findings regarding the effectiveness of CQI and barriers and enablers to CQI implementation. This strong focus on CQI is unsurprising as it aligns with the primary focus of the collaboration. For example, key findings include that CQI, when implemented over time, can improve the quality of care for Aboriginal and Torres Strait Islander peoples. Also, that CQI has been effectively implemented in both clinical and non-clinical health contexts, with tools and processes being successfully adapted to address social determinants of health and health promotion. Additionally, there was agreement that multi-level support, including from policy, leadership and whole-of-organization enables effective CQI as does having the right blend of staff including CQI facilitators, Aboriginal and Torres Strait Islander staff and Aboriginal and Torres Strait Islander leadership. Threats to CQI included barriers to staff retention and training.

There were four instances where more detailed or explanatory information was given in one synthesis type but not another. These were labelled as “moderate agreement.” In all cases, the discrepancies did not provide contradictory information, but rather, one method gave deeper insights into statements posed by another. Betzner et al. [44] likewise reported that discrepancies across methods yielded useful insights rather than contradictory information. For example, the Structured Synthesis findings identified that research has not yet explained variations observed across sites in levels of improvement linked to CQI processes. The Participatory Synthesis went farther, identifying that variations are due to incomplete and problematic implementation of CQI, the top-down selection of CQI issues for study that yield little buy-in from staff, and decoupling of data collection processes from improvement processes.

The discrepancies also provide useful information to guide future research. A finding from the Structured Synthesis described how the CRE-IQI research activities have been successful, in part, due to a long history of collaboration research and relationships that establish shared ways of working, and embedded ongoing evaluation. Yet the Participatory Synthesis identified a need to improve community involvement in CQI and in research, particularly through increasing Aboriginal and Torres Strait Islander ownership and empowerment to identify priority questions and support implementation, translation, and credibility of research findings. There were a few instances where findings statements reflected the opposite perspective of the same issue. For example, the Structured Synthesis identified barriers including low staff capacity for CQI and information technology system use. The Participatory Synthesis framed this issue as a need for better recruitment and retention processes, training, and support for staff involved in CQI.

Each synthesis type produced unique insights, with 21 unique findings statements (Table 2). The greatest number of unique findings came from the Participatory Synthesis (*n* = 13), where, as mentioned above, the insights provided useful information to guide implementation, improvements, or further studies. Examples include that CQI tools and processes aren’t fully utilized because of a disconnect between practice and/or the specific context of the tool or theory of CQI.

**Table 2:** Key findings from CRE-IQI publications and reports (*n* = 78) organized by agreement across synthesis methods

	Rapid Synthesis	Structured Synthesis	Participatory Synthesis
Unique findings	• There is wide variation in the quality of delivery of care between health services and jurisdictions, with a significant proportion of this variation explained by health centre factors rather than patient characteristics • Multidisciplinary networks—such as ‘innovation platforms’—are effective in collective problem solving, building capacity and learning, and fostering system-wide learning and change	• There is a need for health centre capacity building in the use of CQI tools and processes and principles of patient-centred care • Staff capacity for CQI and IT system use is low; CQI is not viewed as a core component of staffs’ work—this is linked to high staff turnover and a lack of leadership • Trusting relationships between staff and clients and between Aboriginal and Torres Strait Islander staff and non-Indigenous staff support staff retention, and quality service delivery • There are successful models of staff capacity building for CQI implementation in a variety of areas including: Health promotion, family wellbeing program, innovation platform, and programs for staff development and wellbeing • CQI processes can improve the quality and use of client record systems • Description of the Innovation Platform concept and its application to the CRE-IQI and Aboriginal and Torres Strait Islander Primary Healthcare. The innovation platform concept can be applied to the CRE-IQI an Aboriginal and Torres Strait Islander Primary Healthcare research network	• CQI tools are not fully used [50] because of a disconnect between the theory of doing CQI, and the realities of practice • Remoteness, population size and governance structure types are not linked to ability to conduct CQI [51], but in our experience, organisational commitment, leadership, funding, and support to develop capacity are linked • Good data (i.e., relevant, reliable) [52, 53] is crucial but must be part of a full cycle, at a whole-of-system level, to drive improvement • CQI will be implemented/look different in each service. Each context is unique [53] • Enablers of CQI are participatory and contextually relevant and responsive approaches [54, 55] • Gaps in follow-up care exist across the full pathway of care [56] because systems are not fit for context; better referral systems are needed [57] and support to increase and retain Aboriginal and Torres Strait Islander staff • Better referral systems and support is required to enable Aboriginal and Torres Strait Islander patients to navigate the health system [57] • “Proper care” and standards of care need to be defined by Aboriginal and Torres Strait Islander people themselves [58] • Navigating the healthcare system is problematic, and patients require more help [58] • There is a need for documentation [59], assessment and support, and action in preventive health [60], health promotion and emotional well-being [61, 62], with health promotion and community-based initiatives that incorporate social determinants of health • Diversity in learning and collaboration is supported by mechanisms that bring diverse groups together [27, 56, 63] to do monitoring [63] and collaboration in writing [64], and it enables dissemination of findings at different levels of the system [65] • Ensure capacity strengthening and succession planning is embedded in research activities/programs [30, 64] • Consistency in reporting of research and research approaches (including value and economic value) is important [64, 66]
Moderate agreement	Availability of IT data systems may drive high-quality care [50]	IT capacity and use in the context of CQI processes and quality data is poor [67–69] because IT systems and training aren’t specific to CQI	IT systems need to be dynamic to respond to needs, high-level/centralized, with increased focus on capacity building for use [56, 60, 70, 71]
	Leadership for CQI is important [72, 73]. Local-level leadership identified as particularly important	Leadership for CQI is important [72, 73], and lack of leadership is a problem	Leadership for CQI is important [72, 73] and lack of leadership is a problem. Aboriginal and Torres Strait Islander leadership identified as critical
		Studies have not yet explained variations in effectiveness observed across CQI studies [51]	Participant insights suggest variations are based on incomplete cycles of CQI, separation of data from improvement activities, and top-down selection of issues to study
		Activities of the CRE-IQI supported its history of a collaboration research network and ways of working, and ongoing evaluation on its functioning	There is a need to improve community engagement in CQI and research to increase Indigenous ownership and empowerment, identify priority questions, support implementation and translation, and the credibility of findings
Strong agreement across synthesis methods	• Consecutive cycles of CQI improve care [46, 55, 61, 68, 74–81] • Policy support enables CQI [82] • There are variations in effectiveness of CQI on improving care [55, 61, 68, 76, 78, 79] • CQI processes have been applied to a range of non-healthcare settings and for a variety of purposes [83, 84] • Reliable, valid CQI tools and research have been developed and adapted to support changes to care for a range of issues [45, 46, 68–70, 72, 85–88] • CQI processes and CQI tools have been successfully applied to other, non-clinical areas of care—including to address social determinants of health and health promotion—and are adaptable for context and content [45, 46] • Multi-level leadership across an organization is needed to support CQI [73, 82, 89] • Retaining staff is critical for skilled teams to engage in CQI [53, 72, 73, 89–91] • Barriers to training and retention include heavy workloads and time pressures; with recommendations from Participatory Synthesis to decrease workloads and focus on hiring Aboriginal and Torres Strait Islander staff [58, 92] • Whole-team, whole-organization support for CQI is an important enabler [73, 82, 89] • Specialized CQI Facilitators support CQI processes, including promoting team dynamics, capacity building and effective use of CQI tools [45, 46, 93, 94] • Having the right blend of staff, including Aboriginal and Torres Strait Islander and local workforces, is an important enabler of CQI [53, 57, 73, 90, 95, 96] • Having Aboriginal and Torres Strait Islander staff, a stable workforce including Aboriginal workers and local workers, and having clear roles and responsibilities of staff are vital to providing care and engaging in CQI [53, 72, 73, 89–91, 96] • Aboriginal and Torres Strait Islander staff and Aboriginal and Torres Strait Islander participation input are critical in driving the quality of care [53, 57, 73, 90, 95] • Follow-up of abnormal results is a high priority for Primary Health Care [62, 71, 96] • Importance of Aboriginal and Torres Strait Islander leadership and ownership of driving quality, CQI and CQI research [30, 97]		

Citations were identified by participants during the Participatory Synthesis as they crafted these statements. As such, they may not fully reflect all the CRE-IQI articles that speak to these issues. Some findings have been edited for clarity. *CQI*  Continuous Quality Improvement, *IT*  Information Technology, *CRE-IQI*  Centre for Research Excellence in Integrated Quality Improvement

## Discussion

We offer an innovative approach to evidence synthesis that actively involves participants in synthesising evidence and generating findings statements themselves. This contrasts with previous studies that engage participant perspectives in parallel to a structured analysis by researchers, or post-synthesis to verify or help disseminate findings [21–25]. Our approach provides a process that explicitly links citations of published research alongside experiential and contextual knowledge of practitioners. We demonstrate that a large quantity of evidence can be synthesised in a relatively short workshop, which is important given that lack of time is one reason cited for not engaging participants in syntheses processes [19]. Further, our analytical comparison of the participatory findings against traditional review approaches—i.e., via a rapid review and a systematic, structured review—underscores the quality and accuracy of the participatory findings, and demonstrates the added value of the participatory analysis. For example, the participatory analysis yielded concrete actions to guide future research to be more participatory while also examining ways to strengthen Aboriginal and Torres Strait Islander involvement in health care. These actions are being implemented within a subsequent, Indigenous-led Centre for Research Excellence (further described below). Our analysis shows that the findings produced in the participatory workshop agreed with findings produced via traditional methods while also generating a greater number of unique findings statements. This adds to previous research which shows that traditional approaches may miss what is important to practitioners [22].

### Strengths, limitations, and lessons learned

This work is embedded in an ongoing collaboration that has an explicitly stated commitment to collaborative and participatory processes. Hence, a history of trustful partnership made this research possible as relationships between participants and our principle-driven commitments would have supported the research/service provider teams to collaborate during the workshop. Future applications should plan to design in mechanisms that promote power sharing. Given the open nature of the collaboration, however, some participants in the process were new to the CRE-IQI. Despite knowing little to nothing about the CRE-IQI’s research, they were able to actively participate and contribute to the process.

Also, because these syntheses were not independent of each other, some of the overlap in findings may be due to knowledge being carried forward (i.e., in the initial construction and grouping of publications). While this possibly constitutes a limitation, we feel it strengthened the final findings statements as they benefited from recursive analyses by multiple people. This perspective reflects that knowledge translation is an active, ongoing activity in which knowledge is constantly created and recreated. Yet the findings statements will not perfectly reflect what participants might have chosen had they read the publications themselves. Some information and/or findings may have been missed where choices were made to package information into a digestible format for the participatory process. Participants raised concerns during the workshop that the synthesis results may only be as good as the details provided in the summaries provided. While true, on the other hand it would not be feasible for participants to read multiple full-length publications during a time-limited workshop. This points to the importance of having a quality control mechanism—for us, it involved providing conversation “hosts” who had read the publications and could provide further insights, and undertaking the comparative analysis described here. Yet the Participatory Synthesis yielded more unique insights than the other synthesis approaches, underscoring its value as a tool to identify findings of relevance to stakeholders.

We were able to validate that all statements generated from the Participatory Synthesis were supported by statements in the publications, even when the publications were not provided to the participants at the workshop. For example, a statement about CQI Tools being adaptable to context and useful for relationship, team and capacity building through skilled facilitation was supported by statements in papers about the use of a systems assessment tool [45] and the development of a health promotion CQI tool [46]. Similarly, Harris et al. [22] also found that stakeholders involved in their systematic review identified key findings that were supported by evidence from other studies, but not those included in the review. One explanation is that traditional synthesis may miss what is most important knowledge for practitioners, thereby further underscoring the need for regular involvement of end-users in evidence synthesis processes [22]. Another is that some participatory designs—like the one we describe here—successfully access collective knowledge that draws on the previous training and experiential application of evidence by practitioners in practice.

Commentary-type publications reflecting on research processes were difficult for participants to summarize. In addition, as previously described, we purposefully excluded descriptive, cross-sectional studies reporting quality improvement patient audit outcomes because we were unsure how they could be further summarized or synthesised in the Participatory Synthesis. Participants did not have the opportunity to review these publications which might have generated findings statements that corresponded with the other synthesis methods. This suggests that our process might work better with some kinds of research—perhaps intervention, and pragmatic interview studies rather than commentaries or highly contextual qualitative analyses (e.g., ethnographies). Given that many of our participants work in clinical contexts, their familiarity with medical research reporting conventions might also have facilitated their engagement. Future studies should examine whether and how this process could be adapted to work in other contexts, with other stakeholder groups, types of research designs and academic outputs.

Each synthesis looked at slightly different subsets of the collaborations’ publications and were conducted for slightly different purposes. But the formats provided us an opportunity to compare and trial approaches used for real-world synthesis purposes rather than artificially set up a lengthy review process that might not feasibly transfer to real-world contexts. Notably, all three were labour intensive approaches, but they may not need to be if the practice of developing approachable research summaries is established as part of a reporting protocol. This last point emphasises the importance of undertaking knowledge synthesis and translation as an ongoing endeavour—not one that begins at the end of a project [47].

Finally, this research occurred within the context of a collaboration with and for Aboriginal and Torres Strait Islander people. While Aboriginal people did participate in the synthesis and as co-authors of this paper, the participatory process was not designed from an Indigenous perspective. The research from the CRE-IQI is intended for both Indigenous and non-Indigenous audiences invested in improving the health and wellness of Indigenous peoples through primary healthcare. Yet it is ultimately meant to have positive impacts on the health and wellness of Indigenous peoples who are, therefore, the primary intended beneficiaries. Best practice in knowledge translation in Indigenous settings places Indigenous peoples as leaders to ensure that translation of research aligns with Indigenous worldviews, and identifies research messages that are defined and desired by Indigenous communities [47]. While the process\es reported here were not Indigenous-led, this is only one of many knowledge translation activities that the CRE-IQI undertook, including processes that were co-designed to be culturally responsive for Aboriginal and Torres Strait Islander communities. These principles, and the insights gained through the work of the CRE-IQI, are informing knowledge translation in the current iteration of the research collaborative in the CRE-Strengthening Systems for Indigenous Health Care Equity (CRE-STRIDE, 2020–2024). CRE-STRIDE is led by Aboriginal and Torres Strait Islander people alongside non-Indigenous allies [26].

### Implications for policy and practice

One benefit arising from the participatory synthesis process that we describe here is that it provides a means for researchers to work alongside other stakeholders to translate research into quality and impactful messages. It demonstrates that processes such as ours can involve stakeholders to contribute policy guidance based on the accurate interpretation of research. In the context of large-scale research collaborations, processes of knowledge translation that effectively engage end-users have yet to be established [48]. We offer a method that can be applied by other collaborations seeking to synthesize a large body of diverse research outputs in a participatory way.

Given that there was strong agreement for most findings, none of the synthesis approaches we trialled appears to generate more accurate findings than another. Yet each yielded ancillary benefits including participation, contextualization, and summative descriptions—which, depending on one’s purpose, may be of interest to future contexts.

Notably, the Participatory Synthesis yielded the most directive and action-focused statements of the three methods. This likely reflects the involvement of practitioners who can contextualize the implications of research in terms of needed systemic changes and improvements, whereas academics may struggle with whether to be neutral providers of evidence or active advocates of change [49]. Collaborative approaches that blend experiential knowledge with research evidence, like the approach we offer here, may help embolden research teams to generate more directive research outputs for guiding policy and directing practice.

The participatory process we used could be easily adapted to large, collaborative research groups working in other subject areas. Much of the preparation work we did involved generating approachable publication summaries that differed from published abstracts in that they focused more on findings than on background and methods (though some details were provided). To aid future processes, authors could provide these plain(er)-language summaries along with their publications during productivity reporting and decrease the amount of preparatory work. We feel that the Structured Synthesis and Rapid Synthesis, though helpful in informing our thinking in this initial design of the participatory review, are not necessary for those wishing to use the participatory process in the future. Nor, we suggest, is the “evidence-checking” step we undertook to link all of the participatory findings to citations for this analysis and our final report given the strong overlap and quality generated from our synthesis design.

However, we are aware of the irony in our advocating for participatory processes whilst also comparing our findings to traditional methods and ensuring that final statements were supported by evidence. The strong preference among our stakeholders for traditional academic knowledge-citing in our purposefully participatory synthesis process begs for reflection. Our experience demonstrates an ongoing tension between academic research communities and non-traditional, innovative approaches to knowledge generation. In part, the purpose of our three-way comparison is to push-back against the assumption that systematically identified and cited publications have a higher value than a combination of academic and experiential knowledge. And conceptually, the accuracy of the participatory findings should be unsurprising. The workshop participants—whether or not they were formally involved in generating any of the publications reviewed—live and work in the context in which the research was generated. In many ways, the published research only captures and codifies knowledge which the practitioners live with and create daily.

## Conclusion

The process we report here will be of interest to other research networks, multi-stakeholder research groups, and other large-scale research producers, across disciplines, who may wish to adapt and test this approach in their contexts. Our experience of involving end-users in a knowledge translation process yielded similar findings to more “traditional” forms of synthesis, but provided contextual information, new insights, and directions for future research. As research collaborations continue to be funded to make impactful systemic change, successful knowledge translation is imperative and more examples of innovations in this area are encouraged.

### Supplementary Information

**Additional file 1.** Findings statements by synthesis approach. List of findings by each synthesis type.

**Additional file 2.** Participatory synthesis activity guide.doc Participatory synthesis protocol. Detailed outline of the participatory synthesis workshop and activities.

**Additional file 3.** Comparison of synthesis outputs.doc Summary of agreement and disagreement among synthesis approaches of CRE-IQI findings. Table reporting results of the cross-synthesis analysis.

## Data Availability

Most data generated or analysed during this study are included in this published article and its supplementary information files. Additional data are available from the corresponding author on reasonable request.

## Abbreviations

CRE-IQI: Centre for Research Excellence in Integrated Quality Improvement
CRE-STRIDE: Centre for Research Excellence Strengthening Systems for Indigenous Health Care Equity
CQI: Continuous Quality Improvement
