# Supplementary material for: Producing knowledge together: a participatory approach to synthesising research across a large-scale collaboration in Aboriginal and Torres Strait Islander health
Source: Health Res Policy Syst. 2024 Jan 3;22:3. doi: 10.1186/s12961-023-01087-2 (PMC10765661; doi:10.1186/s12961-023-01087-2)
Supplement: Supplementary file 1 — Additional file 1. Findings statements by synthesis approach. List of findings by each synthesis type. [file 12961_2023_1087_MOESM1_ESM.pdf]

Appendix 1: Findings Statements from by synthesis approach

Findings from the Structured Synthesis, organized by CRE-IQI Aim

Aim 1: Using, refining or developing a clinical audit and other tools for application in QI and PHCs

- Six tools developed for applied CQI use and CQI research
- 42 articles report data collected from CQI tools, and/or describe adapting CQI tools for application in research
- There is a need for capacity building in using CQI tools and processes and IT systems,
- CQI tools support changes to care

Aim 2: Examine CQI/QI approaches and processes, and interventions (including barriers/facilitators), including for example, support for CQI data use and governance

- Evidence that CQI over time has improved care across a range of health topics
- Consecutive cycles of CQI are associated with improvements
- But in some CQI studies, there were variations in improvement across services, a plateauing effect, or no effect observed. Subsequent studies have not yet explained variations
- Barriers/enablers of CQI can be organized into three levels
    - Micro – Policy level support
    - Meso – whole team approaches, training, regular assessment against KPIS, supported QI
    - Micro – facilitators are important to supporting CQI and using tools effectively
- Staff capacity for CQI and IT system use is low, CQI not viewed as core component of work – this is linked to staff turnover, and lack of leadership
- CQI processes are being translated to policy- and regional-level action, other non-clinical issues and are showing promise

Aim 3: Examine/Refine QI data systems to make effective, efficient and reliable use of data for quality improvement (including, e.g. electronic clinical information systems, and indicator data that services are required to report)

- Clinical IT systems improve care by supporting best practice and addressing priority-evidence gaps
- IT systems lack ability to extract meaningful data for improvement including planning and evaluation, lack uniformity across services, and are not responsive to user needs
    a. In one study, paper records had better data and use given barriers to electronic systems
- Staff capability and technical difficulties
- CQI processes can improve the quality and use of client records systems

Aim 4: Identify and/or develop and evaluate strategies and resources to increase capacity within the Indigenous PHC workforce and leadership in skills, knowledge and attributes that evidence indicates will support large-scale continuous improvement in Indigenous quality of care

- Importance of Indigenous leadership, and the critical role that Indigenous people play in delivering patient-centered, quality healthcare
- Retaining and recruiting appropriate staff is a critical problem that has a knock-on effect for addressing all subsequent barriers
- Training and retention barriers for staff include heavy workloads and time pressures
- Need the right staff, including an appropriate blend of Indigenous and non-Indigenous staff, specialists, and Aboriginal Health Workers

Published in: Conte, K. P., Laycock, A. F., Bailie, J., Walke, E., Onnis, L., Feeney, L., Langham, E., Cunningham, F. C., Matthews, V., & Bailie, R. S. Producing knowledge together: a participatory approach to synthesising research across a large-scale collaboration in Aboriginal and Torres Strait Islander health. *Health Research Policy and Systems*. https://doi.org/10.1186/s12961-023-01087-2

- Trusting relationships between staff and clients, and between Indigenous and non-Indigenous staff support retention, and service delivery
- Successful models of staff capacity building include in: Health promotion, family wellbeing program, Innovation platform, and programs for staff development and wellbeing

Aim 5: Monitor, describe and/or evaluate Innovation Platforms as a mechanism for large-scale change in primary healthcare

- Introduces the Innovation Platform concept and applies it to the CRE-IQI and Indigenous Primary Healthcare
- Papers outline how the CRE-IQI works including the collaborative network the precedes is supported by CRE-IQI; implementation of the collaborative ways of working, and plans for research and evaluation on the CRE itself.

Findings from the Participatory Synthesis, organized by categorization for workshop

CQI Processes

- CQI implemented over consecutive cycles improves care processes
- But in some circumstances, there is variation in delivery of CQI between services, a plateauing effect, and no effect- perhaps due to incomplete cycles, top-down selection of issues, separation of data collection from improvement activities (i.e., implementation failure!)
- Potential new applications for CQI in other sectors and for other purposes (i.e., evaluation)
- Remoteness, population size and governance do not impact ability to conduct CQI, but in our experience, organisational commitment, leadership, funding, and support to develop capacity does
- Enablers of CQI are participatory & contextually relevant and responsive approaches

CQI Tools and IT Systems

- CQI tools have been developed and proven reliable and valid for a range of issues, but aren't fully used because there is a disconnect between the theory of doing continuous quality improvement, and the realities of practice
- CQI Tools (including health promotion and SAT tools) are adaptable to context are useful for relationship, team and capacity building through skilled facilitation
- Systems - need flexible and dynamic systems with centralized IT systems and high-level databases, increase recording and monitoring and training to do it.
- Although IT systems can support improved practice, generally, IT capacity and use in the context of CQI processes and quality data is poor because IT systems and training aren't specific to CQI

CQI Context

- Stable workforce, including Aboriginal & local workforce, and stable systems enable teams to engage in CQI
- Good data (ie relevant, reliable) is crucial but must be part of a full cycle, at a whole-of-system level, to drive improvement
- A whole-of-organisation approach supported by leadership at all levels is needed to support CQI
- CQI will be implemented/look different in each service. Each context is unique

Priorities in Primary Health Care

- Proper care needs to be defined by Aboriginal people
- Gaps in follow-up care exist across the full pathway of care because systems aren't fit for context
- Need help navigating the healthcare system

- Need better referral systems and support, need to reduce turnover and increase Aboriginal staff
- Holistic teams, with Aboriginal staff, with skills and clear roles (and responsibilities; high turn-over=knowledge loss. Need more Aboriginal staff, but they are decreasing
    - Recommendation: decrease workloads, reduce staff turnover, increase Aboriginal staff
- There is a great need to follow-up Abnormal results and with people whose symptoms are worsening
- Need documentation, assessment and support, and action in preventive health, health promotion and emotional well-being, with health promotion, community-based initiatives that incorporate social determinants of health

Research Approaches

- Need for Indigenous control/ownership/leadership via processes, funding, principles of working = commitment and effort
- Diversity in learning and collaboration is supported through mechanisms to bring diverse groups together, monitoring, collaborative writing and enables dissemination of findings at different levels of the system
- Ensure capacity strengthening and succession planning is embedded in research activities/programs
- Improve community engagement in CQI and research - via mutually respectful relationships - to increase ownership and empowerment, identify priority questions, and support implementation and translation, and credibility of findings
- Consistency in reporting of research and research approaches (including value and economic value) is important

Findings from the Rapid Synthesis, as reported in Bailie et al. (2018)

- Sustained use of CQI is associated with improved adherence to best practice care, particularly when backed by higher level policy and regional-level support.
- CQI has been successfully applied in Australian Aboriginal and Torres Strait Islander and PHC settings as well as in non-clinical areas of work with a direct bearing on the social determinants of health.
- For CQI initiatives to have maximum effect in improving the quality of health service delivery to Aboriginal and Torres Strait Islander people it is important to support local-level leadership and decision making in this area.
- There is a wide variation in the delivery of care between health services and jurisdictions, with a significant proportion of this variation explained by health centre factors rather than patient characteristics.
- The design of the delivery system – clinic infrastructure, staffing profile and the allocation of roles and responsibilities – is vital to the provision of evidence-based care.
- The availability and use of clinical information systems, committed leadership support for CQI processes, and strong Indigenous participation in the health workforce drive the delivery of high-quality care.
- A lack of follow-up of abnormal results across a number of areas of care has been identified as a high priority to address.
- Aboriginal and Torres Strait Islander leadership and participation in CQI research is key to aligning research and implementation with the priorities of Indigenous PHC services and communities.
- Access to accurate and timely data across the full scope of best practice for PHC is required for both community and data-driven action.
- There are critical gaps in the collecting and recording of client data in clinical information systems, with an identified need for more training in system use.
- Multidisciplinary networks – such as 'innovation platforms' – are effective in collective problem solving, building capacity and learning, and fostering system-wide learning and change.

References

Bailie, J., Laycock, A., Harkin, K., Conte, K. P., & Bailie, R. (2018). *CRE-IQI Year 4 Review Progress Report 2018: Strengthening the Health System through Integrated Quality Improvement and Partnership.* . U. C. f. R. Health.
