# Supplementary material for: Producing knowledge together: a participatory approach to synthesising research across a large-scale collaboration in Aboriginal and Torres Strait Islander health
Source: Health Res Policy Syst. 2024 Jan 3;22:3. doi: 10.1186/s12961-023-01087-2 (PMC10765661; doi:10.1186/s12961-023-01087-2)
Supplement: Supplementary file 2 — Additional file 2. Participatory synthesis activity guide.doc Participatory synthesis protocol. Detailed outline of the participatory synthesis workshop and activities. [file 12961_2023_1087_MOESM2_ESM.pdf]

Appendix 2

PARTICIPATORY SYNTHESIS PROTOCOL

Workshop Aims:

1) Using summaries of the research produced in each of the CRE-IQI aims, and a facilitated process, identify the key/most meaningful research findings
2) Reflect on and capture knowledge produced alongside the research (e.g. experiential knowledge) that may not be captured in the published research reports
3) Describe and reflect on the effectiveness of the facilitated process to elicit key findings

(including how well a participatory synthesis and workshop is able to capture findings and bring diverse perspectives of stakeholders to the synthesis and interpretation process)

WORKSHOP OUTLINE

Duration	Activity	Purpose	Resources
During Registration	**Sign Up for Group Discussion**		Sign up sheets
**2.5 Hours**			
	**PART 1**	**‘Looking back on 4+ years of CRE-IQI research’**	
20 mins	**Introduction**<br>• Rationale<br>• Intended outcomes (Research Translation for policy and services, evidence base for grants)<br>• Explain the 2-stage process<br>- what/who stage 1 involved<br>• Workshop aims<br>• Workshop processes/format<br>• Introduce table leaders and group work	To introduce aims, explain task and process	Power Point

Published in: Conte, K. P., Laycock, A. F., Bailie, J., Walke, E., Onnis, L., Feeney, L., Langham, E., Cunningham, F. C., Matthews, V., & Bailie, R. S. Producing knowledge together: a participatory approach to synthesising research across a large-scale collaboration in Aboriginal and Torres Strait Islander health. *Health Research Policy and Systems*. https://doi.org/10.1186/s12961-023-01087-2

<table>
<tr><td></td><td>Ground Rules
Ethics paperwork</td><td></td><td></td></tr>
<tr><td>10 mins</td><td>Get in Groups and Introductory Reflection
Each person take a moment to write on sticky note:
What do you think has been learned about your topic? Ie. what do you expect to be the findings?</td><td>Start thinking about the topic, consider assumptions/mental models that we carry about the topic</td><td>Sticky Notes and markers/pens</td></tr>
<tr><td>20 mins (10 minutes per article)</td><td>Table work
What are the findings?
Form groups of 2 or 3 people. Each group should take 2-3 articles.
Using paper provided – discuss what you think are the main or most important findings as related to your topic. Using the paper provided, write down the main findings - use a different piece of paper for each finding
Rank the findings in terms of what you think are most interesting or important</td><td>Familiarise participants with the published research outputs

Summarise research findings</td><td>Collated findings - sorted under the research aims/modified categories

Handout Part 1</td></tr>
<tr><td>30 mins
(3 min per article)</td><td>Identify Main Findings
One group at a time, give a brief description of the study. Share the 1 or 2 main findings you decided selected as most interesting or important, and explain why you chose them.
Take turns until all articles have been discussed. You should now have the main findings laid out on butchers’ paper. - organize them into categories; If any of the main findings are repeats, put them together.<ol type="a">
LABEL each category - What is this group of findings about?</td><td>Identify priority or key findings to establish meaning and significance in context of PHC QI

To encourage prioritisation and reflection about the ‘most significant’ (there can be a few most significant, but there are likely some that are not as…)

WHAT are the most important findings?</td><td></td></tr>
</table>

	WOULD BE GOOD TO DISCUSS WHY HERE<br>3. Take a look at the remaining findings that didn't get discussed. Are there any pressing or remaining issues that should be added to these groups? Add them now	To relate project level findings to our task of identifying findings across multiple projects	
20 mins	**REFRESHMENT BREAK**		
10 mins	**Surfacing Experiential Knowledge**<br>1. IN SILENCE - take a look at the work you've done, the categories and their descriptions<br>2. Consider, what else do you know about this issue/topic from your personal/professional experience helps you make sense of or explain why this finding is meaningful or important?<br>3. Write this down on a STICKY NOTE and place it next to the category	Bring in experiential knowledge to help explain/interpret the value of the findings	STICKY NOTES
20	**What is Meaningful About These Findings?**<br>1. For each category, discuss:<br>a. What are the findings in your category telling you about your topic? (ie what insights have we gained about "IT systems and data", or "CQI processes"?)<br>b. What about this "category" is meaningful or important?	WHY are these important?	Materials for recording conversations: recorders<br><br>Butchers paper and pens<br>OR<br>A4 paper

	c. Reflect the role of experiential knowledge<br>d. Are any of the categories you've identified related to each other? Describe this relationship and consider what additional insights this gives you about your topic.		Table leader<br>Scribe
10	**Develop Summary Statements**<br>Develop 1 - 3 statements to summarise the main learnings/ insights<br><br>Write each statement on separate piece of butchers paper	Summarize 'key findings' in 1-3 statements	A4 Sheets - 1 statement per sheet
**1.5 hours**	**PART 2 - DAY 2**	**'Looking ahead to build on our experience and impact'**	
5	**Reflection**<br>Referring back to your sticky note (what did you think you would learn) write down (new sticky? On back?) what you learned/surprised about etc	Reflect on process, on key learnings from this activity	
10 min each, about 40 min total	Whole group<br><br>Post the summary statements around the room.<br><br>1. Explain the focus of this group of articles and/or reports (e.g. improvement priorities, CQI processes). CONVERSATION HOSTS TO STAY WITH THEIR BUTCHER'S PAPER TO GIVE SUMMARY/RECAP<br><br>2. 'Round robin' where people go from table to table to consider:<br>● Is there anything MISSING in your knowledge of CRE-IQI work that could	To gain additional insights from diverse perspectives<br><br><br><br><br><br><br><br><br>Reflect on the research question. Build understanding and interpretation if findings.	Write statements using MARKERS in large print, or on butcher's paper

	be added/give more insight to this finding? What is that insight?<br>• What do you know about this topic/findings that explains its importance<br>• *Potentially, could ask about next steps? Since this is about looking forward?*<br>• add to findings and/or refine interpretations<br>• add to understanding of how they are meaningful<br><br>Whole group discussion:<br>• To what extent do CRE-IQI <u>research findings and approaches</u> reflect the stated aims and principles of the CRE-IQI?<br>• How do they align the cross-cutting work programs?		
20 mons	Whole group reflection on outcomes and process.<br><br>How was this useful/not useful for synthesizing the research findings?<br><br>Did the collaborative interpretation process add value to findings and how/ how not?<br>…as a learning process?<br><br>**Where to from here? Planning for implementation and impact**<br>• Using outcomes in policy pitch Masterclass<br>• Developing research translation messages and products for other audiences	To reflect on the value of the process and its outcomes – usefulness and learnings<br><br>To identify options for use and next steps	Whiteboard to document

	• Knowledge base for functioning of Innovation Platform<br>• Articulated CRE findings to support grant development<br>• How will we describe and share this innovative process		
