# Supplementary material for: Producing knowledge together: a participatory approach to synthesising research across a large-scale collaboration in Aboriginal and Torres Strait Islander health
Source: Health Res Policy Syst. 2024 Jan 3;22:3. doi: 10.1186/s12961-023-01087-2 (PMC10765661; doi:10.1186/s12961-023-01087-2)
Supplement: Supplementary file 3 — Additional file 3. Comparison of synthesis outputs.doc Summary of agreement and disagreement among synthesis approaches of CRE-IQI findings. Table reporting results of the cross-synthesis analysis. [file 12961_2023_1087_MOESM3_ESM.pdf]

Appendix 3: Summary of agreement and disagreement among synthesis approaches of CRE-IQI findings

		Organizing Categories for Cross-Synthesis Analysis				
	N	Effectiveness of CQI	Barriers and Enablers of CQI	Primary Health Care	Data and IT Systems	Research Approaches
Number of Findings (total)	78	18	21	21	11	7
Systematic Synthesis	26	7	8	5	4	2
Participatory Synthesis	35	8	10	10	3	4
Rapid Synthesis	17	4	2	6	4	1
3-way agreements	9					
2-way agreements	15					
Unique Findings	21					
Strong Agreement Between Synthesis Types (total)	20					
Agreement across all three	7	2	0	3	2	0
Agreement with Systematic and Participatory	7	2	5	0	0	0
Agreement with Participatory and Rapid	4	1	1	1	1	0
Agreement with Systematic and Rapid	2	1	0	0	1	0
Moderate Agreement Between Synthesis Types (total)	4					
Moderate Agreement across all three	1	1	1	0	0	0
Moderate Agreement with Systematic and Participatory	2	1	0	0	0	1
Moderate Agreement with Participatory and Rapid	1	0	0	1	0	0
Moderate agreement with Systematic and Rapid	0	0	0	0	0	0
Unique Findings by Systematic Type (total)	21					
Unique to Systematic	6	1	2	1	1	1
Unique to Participatory	13	1	4	5	0	3
Unique to Rapid	2	0	0	1	0	1

Published in: Conte, K. P., Laycock, A. F., Bailie, J., Walke, E., Onnis, L., Feeney, L., Langham, E., Cunningham, F. C., Matthews, V., & Bailie, R. S. Producing knowledge together: a participatory approach to synthesising research across a large-scale collaboration in Aboriginal and Torres Strait Islander health. *Health Research Policy and Systems*. https://doi.org/10.1186/s12961-023-01087-2
